# Generating Correlation Matrices Based on the Boundaries of Their Coefficients

**DOI:** 10.1371/journal.pone.0048902

**Published:** 2012-11-12

**Authors:** Kawee Numpacharoen, Amporn Atsawarungruangkit

**Affiliations:** 1 Financial Product Development, Kasikorn Securities, Bangkok, Thailand; 2 Department of Mathematics, Faculty of Science, Mahidol University, Bangkok, Thailand; 3 College of Medicine, Rangsit University, Bangkok, Thailand; Università del Piemonte Orientale, Italy

## Abstract

Correlation coefficients among multiple variables are commonly described in the form of matrices. Applications of such correlation matrices can be found in many fields, such as finance, engineering, statistics, and medicine. This article proposes an efficient way to sequentially obtain the theoretical bounds of correlation coefficients together with an algorithm to generate n 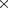 n correlation matrices using any bounded random variables. Interestingly, the correlation matrices generated by this method using uniform random variables as an example produce more extreme relationships among the variables than other methods, which might be useful for modeling complex biological systems where rare cases are very important.

## Introduction

Many important properties of financial models, engineering problems, and biological systems can be represented as correlation matrices, which describe the linear relationships among variables. It is not always the case that these correlation matrices are known; therefore, correlation matrices are an integral part of simulation techniques for solving or analyzing problems in, for example, signal processing [1], portfolio selection [2], factor analytic research [3], genetic modeling [4], and neuroscience [5].

To create a correlation matrix, it is important to ensure that it is valid, meaning that the matrix must be symmetric and positive semi-definite, with the unit diagonal and other elements in the closed interval [−1, 1]. On the contrary, an invalid correlation matrix is one in which assets or variables cannot be correlated according to the specified relationship. The simplest method for constructing a correlation matrix is to use the rejection sampling method, which generates correlation coefficients using uniform random variables in the closed interval [−1, 1]. Subsequently, we check whether the matrix is semi-definite and, if not, another correlation matrix is generated. This procedure is repeated until a valid matrix is obtained. Further details of rejection sampling will be described later in this article. For a low-dimensional matrix, it is relatively easy to use rejection sampling, but when the dimension is greater than or equal to four, the chance of finding a valid correlation matrix becomes very low. However, the number of variables in physical or economic systems is normally considerably greater than four, and so the rejection sampling method is considered inefficient for the large-scale construction of correlation matrices.

Instead, for large-dimensional problems, there are several techniques for generating a correlation matrix. These can be classified, based on the relevant objectives or constraints, as follows:

1. Generating of a correlation matrix with predetermined eigenvalues and spectrum [6], [7], [8];
2. Generating of a correlation matrix with a given mean value [9];
3. Generating of a correlation matrix based on a random Gram matrix [10]; and
4. Generating of a correlation matrix in which each correlation coefficient is distributed within its boundaries [11].

This article focuses on the fourth method presenting an efficient algorithm to calculate the theoretical boundaries of correlation coefficients without the use of optimization techniques. Instead, the theoretical boundaries of each correlation coefficient are calculated from the mathematical structure of the correlation matrix constructed by hypersphere decomposition [12]. Although the theoretical work conducted in [11] is similar to the methodology presented here, its primary technique is the optimization approach, whereas our work uses a non-optimization technique. In addition, the sequence for computing the boundaries of each correlation coefficient is heavily reliant on the concept of adjusting the correlation matrix [13] and its boundaries [14]. After finding the theoretical bounds, we present the techniques for generating a correlation matrix.

## Methods

### Valid correlation matrix

It is important to have a common understanding of the definition of a valid correlation matrix. Such a matrix conforms to the following properties:

1. All diagonal entries must be equal to one;
2. Non-diagonal elements consist entirely of real numbers in the closed interval [−1, 1];
3. The matrix is symmetric; and
4. The matrix is positive semi-definite.

The first three requirements are relatively easy to satisfy. However, the final property of being positive semi-definite requires all eigenvalues to be greater than or equal to zero.

Interestingly, a valid correlation matrix (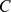) can be constructed using a method proposed in [12] in terms of trigonometric functions. The correlation matrix then becomes a function of angles (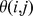), which finally gives an efficient way of computing the correlation matrix boundaries without using an optimization method. According to [12], the valid correlation matrix can be described as:
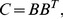
(1)

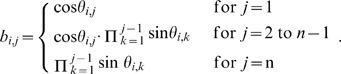
(2)Generally, 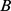 is a square matrix with 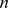 dimensions whose elements are represented by the 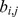 in (2). As explained in [15], (2) can be simplified by setting 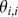 to zero for all i. 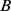 then reduces to a lower triangular matrix, and:
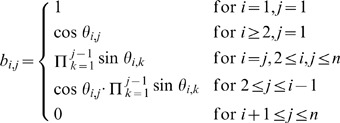
(3)As a result, 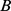 can be expressed as
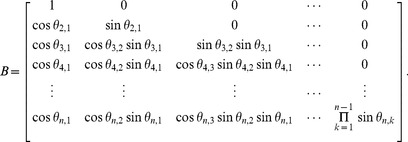
(4)


It is evident from (4) that matrix 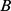 depends solely on 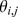, which is called the correlative angle. The square matrix of correlative angles (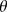) is defined as:
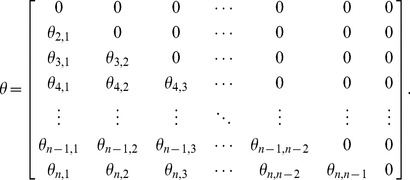
(5)


Thus, a valid correlation matrix can be calculated if the correlative angle matrix (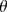) in (2.5)is known.

#### Example 1

Let us assume that the four-dimensional correlative angle matrix is:
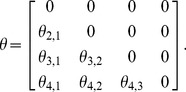
(6)


The matrix 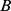 can then be expressed as:
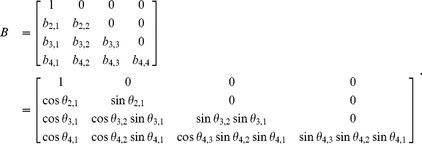
(7)


Finally, the correlation matrix is:
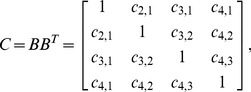
(8)where
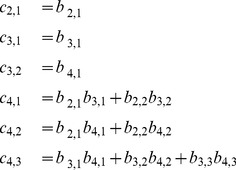
(9)which can be written in terms of the correlative angles as
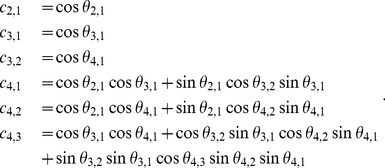
(10)


### Boundaries of the correlation coefficients

As shown in (6) to (10), a valid correlation matrix can be constructed from the matrix 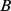, and the elements in 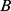 are determined by the correlative angles. Consequently, we can determine which elements of 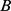 are impacted by changes to the correlative angle in a four-dimensional correlation matrix, from which two important aspects can be inferred:

1. Correlation coefficients in the first column (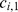) depend solely on 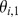.
2. Other correlation coefficients (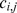) for 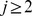) can be calculated if 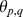 are given, where 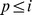 and 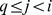.

Because all 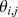 are in the closed interval [0, 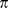], the sine functions will produce non-negative values, whereas the cosine functions will output values in the range [−1, 1]. Using the correlation coefficients in (10) as an example, it is straight forward to conclude that the boundaries of each correlation coefficient (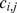) can be calculated by setting 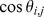 to −1 or 1. Moreover, the boundaries require only 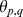 where 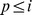 and 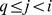, except for 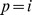 and 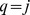 (although not every 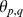 is required), as shown in Table 1. As a result, if 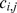 lies within its boundaries and the required 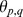 are given, 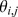 can be calculated by (11).
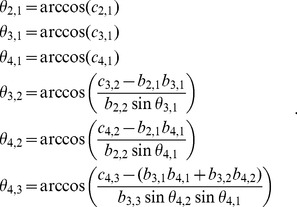
(11)


**Table 1:** Boundaries of each correlation coefficient in a 4
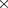
4 matrix.

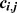	Lower bound	Upper Bound	Required 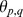
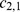	−1	1	No
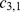	−1	1	No
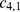	−1	1	No
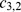	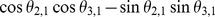	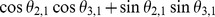	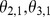
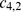	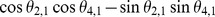	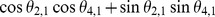	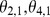
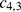	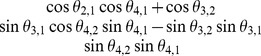	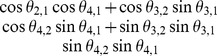	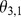 , 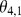 , 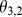 , 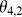

The same logic can easily be applied to higher-dimensional correlation matrices, albeit that longer formulas and computational procedures are obtained.

### Algorithm for constructing a random correlation matrix

This section describes an algorithm to obtain a correlation matrix by sequentially computing the boundaries of each correlation coefficient, as described in earlier section, and generating uniform random variables (other bounded distributions can always be substituted) within these boundaries. Nevertheless, it is important to note that no optimization is needed to calculate the boundaries of each correlation coefficient. This non-optimization approach is the major difference between our work and that from presented in [11]. Let [0, 1] be the strictly lower triangular matrix of uniform random variables in the closed interval [0, 1], 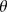 be the strictly lower triangular matrix of correlative angles, and 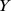 and 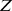 be the strictly lower triangular matrix of lower and upper bounds of the correlation coefficients, respectively. The four-step algorithm for constructing an 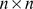 correlation matrix is then:

Step 1: Calculate correlation coefficients in the first column

- For 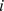 = 1, 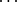, 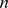, set 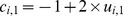, 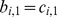, and extract 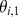.
- For 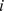 = 2, 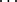, 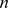, set 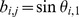 for 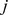 = 1, 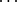, 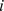.

Step 2: Calculate the remaining correlation coefficients from the third row to the last row and from the second column to the last column of each row.

For 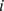 = 3, 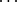, 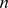


For 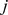 = 2, 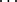, 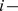
1

- Calculate the lower bound (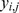) and upper bound (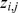) of each correlation coefficient.
  The method for calculating these boundaries is explained in the earlier section. Please see Table 1 for an example of the upper and lower bounds using a four-dimensional correlation matrix.
- If 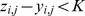, then 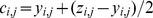. Otherwise, using 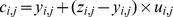.
  During our large numerical experiment, numerical instability occurs when the boundary gap (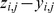) becomes very small. As a result a threshold factor (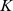) is introduced. This reduces instability by forcing every correlation coefficient with a boundary gap of less than 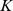 to be centered within its boundaries. Larger value of 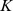 will produce a more stable system, but imply less randomization in the 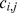.
- Extract 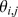 using similar formulas to those shown in (11).

End

End

- Create a symmetric correlation matrix with unit diagonal elements based on all generated correlation coefficients.

**Figure 1:**
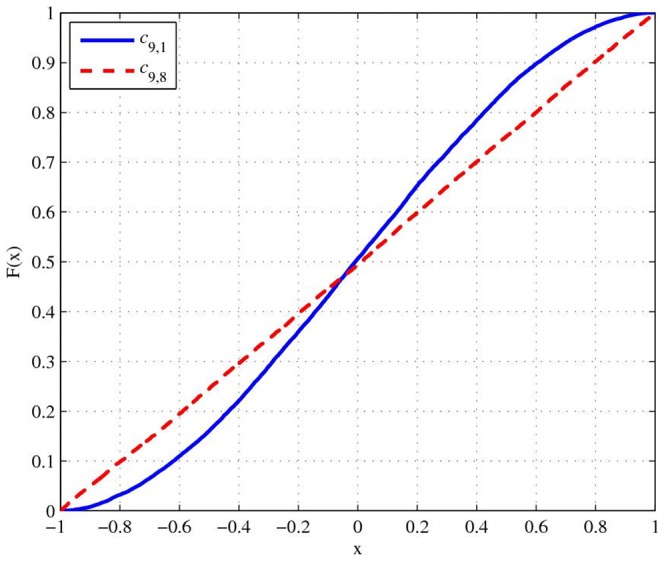
CDF from the proposed algorithm without random reordering.

**Figure 2:**
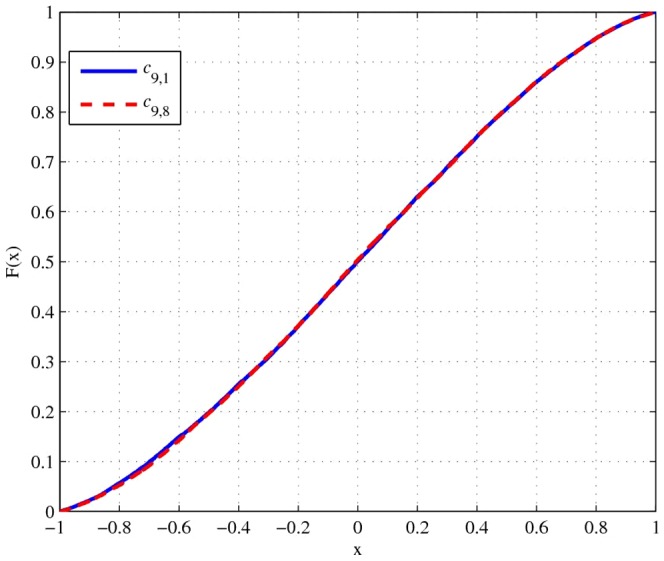
CDF from the proposed algorithm with random reordering.

Step 3: Randomly reorder the correlation matrix. The underlying concept of this step is to ensure that every correlation coefficient is equally distributed. Without this step, the cumulative distribution function (CDF) of correlation coefficients will not be the same (see Figure 1). After applying random reordering, the CDF of the same correlation coefficients will be almost identical, as displayed in Figure 2.

Step 4: Check the validity of the correlation matrix. Even though the above steps should theoretically generate a valid correlation matrix, in some cases numerical instability can still occur. We can detect two major causes of instability: Firstly, K is too low relative to the dimension of matrix; Secondly, generated correlation coefficients are very close to the boundaries. Based on our experiments, in which 1 million 100
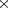
100 correlation matrices were generated with 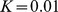, there is only 0.0167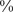 (or 167 matrices) probability that an invalid correlation matrix will occur. Although the probability of an invalid matrix is very small, it is non-zero. That is why this step is necessary, to ensure that invalid correlation matrices will be rejected. The two basic procedures of this step are:

1. Check the minimum eigenvalue. If it is negative, the correlation matrix is invalid. Otherwise, the correlation matrix is valid.
2. Reject the invalid correlation matrix, and regenerate the correlation matrix by returning to step 1

In addition, from (1) to (4), we can generate a valid correlation matrix directly from random sample of correlative angles. Unfortunately, based on our experiment, this direct method is not numerically stable. As a result, one may not be able to use the matrix generated from this method in some applications. Thus, we believe that our new algorithm is superior in terms of numerical stability.

#### Example 2

For a five-dimensional correlation matrix, let us assume that the uniform random matrix 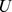 described in step 1 of the algorithm is:
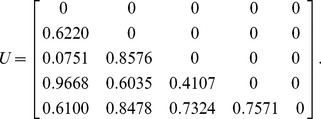
(12)


The lower-bound matrix 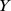, upper-bound matrix 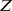, and correlation matrix 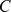 (before being randomly reordered) can then be generated as follows:
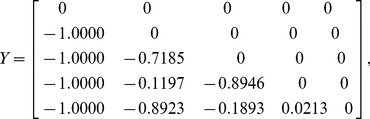
(13)

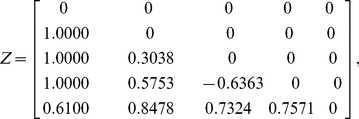
(14)

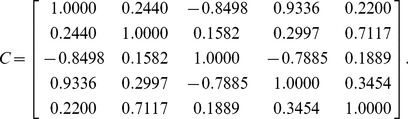
(15)


As the minimum eigenvalue of 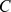 in (15) is 0.00510, the correlation matrix is positive semi-definite. This confirms that 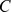 is a valid correlation matrix.

## Results

All numerical tests in this study were conducted with MATLAB 7.8.0 (R2009a) on an Intel(R) Core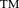 2 Duo CPU T6600 at 220 GHz with 3.50 GB of RAM. The computational performance and probability distribution function (PDF) of the proposed algorithm (NA) with 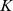 = 0.01 was evaluated and compared with the following two algorithms:

1. Rejection sampling method (RS)The rejection sampling method uses uniform random variables in the closed interval [−1, 1] to represent each correlation coefficient in the symmetric correlation matrix. The correlation matrix will be rejected if it is invalid.
2. Randcorr function of MATLAB (RC)This algorithm is implemented as a MATLAB function, and is based on the work in [6] and [7].

The MATLAB code for the NA algorithm (denoted as RandomCorr) is available at http://www.mathworks.com/matlabcentral/fileexchange/loadFile.do?objectId=37804. The following MATLAB code was used to generate the correlation matrices (*C*) in the RS algorithm and to check their validity: C = tril(−1+2*round(rand(n,n)*10∧8)/(10∧8),−1);C = C+C′+ eye(n);p = min(eig(C));. And the following MATLAB code was used to generate the correlation matrices (*C*) in the RC algorithm and to check their validity: C = gallery(‘randcorr’,n);p = min(eig(C));.

### Computational performance

The computational performance of each algorithm is primarily measured by the expected run time (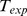), which can be calculated from the average run time (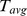) divided by the probability of the generated correlation matrix being valid (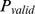). 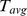 includes the time taken to construct the correlation matrix and calculate the minimum eigenvalue. The performance summary of the three algorithms over 1 million simulations is illustrated in Table 2.

**Table 2:** Comparison of computational performance.

	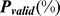			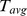 (ms)			T*_exp_* (*ms*)		
n	NA	RS	RC	NA	RS	RC	NA	RS	RC
2	100	100	100	0.0492	0.0149	0.3819	0.0492	0.0149	0.3819
3	100	61.678	100	0.0710	0.0185	0.4720	0.0710	0.0300	0.4720
4	100	18.2341	100	0.0900	0.0204	0.5688	0.0900	0.1121	0.5688
5	100	2.1723	100	0.1164	0.0229	0.6521	0.1164	1.0532	0.6521
6	100	0.1009	100	0.1501	0.0254	0.7472	0.1501	25.19	0.7472
7	100	0.001	100	0.1827	0.0385	0.8567	0.1827	3,849.7	0.8567
8	100	0	100	0.2306	0.0321	0.9669	0.2306	Inf.	0.9669
9	100	0	100	0.2804	0.0355	1.1653	0.2804	Inf.	1.1653
10	100	0	100	0.3304	0.0404	1.2686	0.3304	Inf.	1.2686
11	100	0	100	0.4039	0.0449	1.2318	0.4039	Inf.	1.2318
12	100	0	100	0.4586	0.0485	1.3230	0.4586	Inf.	1.3230
13	100	0	100	0.5513	0.0546	1.4448	0.5513	Inf.	1.4448
14	100	0	100	0.6138	0.0589	1.5067	0.6138	Inf.	1.5067
15	100	0	100	0.6987	0.0647	1.6531	0.6987	Inf.	1.6531
16	100	0	100	0.7788	0.0785	1.7076	0.7788	Inf.	1.7076
17	100	0	100	0.8957	0.0811	1.8294	0.8957	Inf.	1.8294
18	100	0	100	1.0106	0.0873	1.9429	1.0106	Inf.	1.9429
19	100	0	100	1.0990	0.0907	2.0996	1.0990	Inf.	2.0996
20	100	0	100	1.2094	0.0974	2.2008	1.2094	Inf.	2.2008
21	100	0	100	1.3406	0.1051	2.2840	1.3406	Inf.	2.2840
22	100	0	100	1.4722	0.1132	2.3952	1.4722	Inf.	2.3952
23	100	0	100	1.6269	0.1217	2.5304	1.6269	Inf.	2.5304
24	100	0	100	1.7746	0.1296	2.6631	1.7746	Inf.	2.6631
25	100	0	100	1.9446	0.1393	2.7386	1.9446	Inf.	2.7386
26	100	0	100	2.1356	0.1492	2.8582	2.1356	Inf.	2.8582
27	100	0	100	2.2533	0.1585	2.9899	2.2533	Inf.	2.9899
28	100	0	100	2.4576	0.1689	3.0942	2.4576	Inf.	3.0942
29	100	0	100	2.6411	0.1806	3.2981	2.6411	Inf.	3.2981
30	100	0	100	2.8306	0.1904	3.4048	2.8306	Inf.	3.4048
35	100	0	100	3.9381	0.3315	4.0185	3.9381	Inf.	4.0185
40	100	0	100	5.3749	0.3971	4.7135	5.3749	Inf.	4.7135
45	100	0	100	6.8185	0.5067	5.7925	6.8185	Inf.	5.7925
50	100	0	100	8.5822	0.6172	8.5822	8.5822	Inf.	6.9464

Note: Inf. denotes infinity.

With a 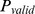 score of 100% in all cases, both NA and RC algorithms are evidently stable. Moreover, the RC algorithm has the fastest expected run time when the dimension exceeds 35, although the RS algorithm is the fastest for dimensions of two and three. However, the RS method then becomes slower than the NA algorithm when 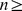
4, and slower than RC for 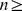
5. Even worse, the RS method cannot generate a valid correlation matrix for dimensions larger than seven, mainly due to the significant drop in Pvalid. Hence, the RS method is not very useful in practice. For dimensions from 4–35, the NA algorithm outperforms RS and RC in terms of expected run time.

### Probability distribution function

**Figure 3:**
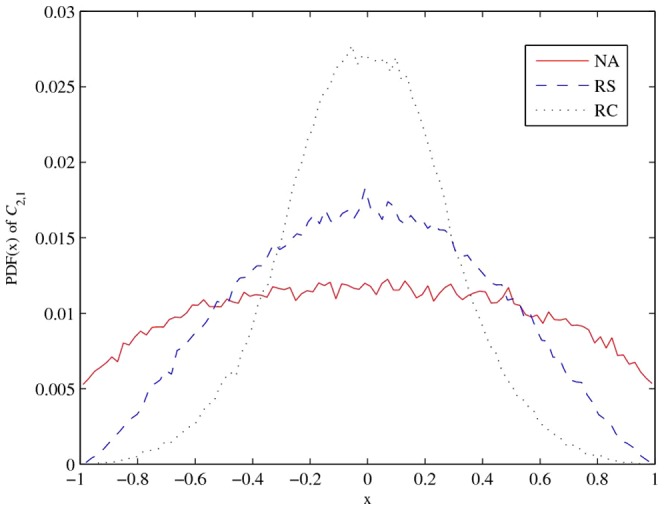
PDF of correlation coefficient ( 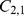
 **).**

**Figure 4:**
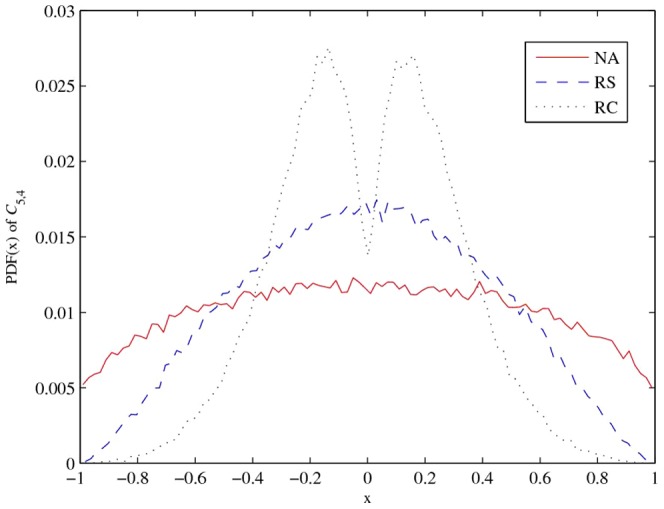
PDF of correlation coefficient ( 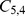
 **).**

To compare the PDF of the coefficients of correlation matrices, 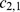 and 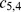 are drawn from 100,000 valid 5
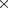
5 correlation matrices constructed by the above algorithms. Comparing Figures 3 and 4, we can clearly see that the correlation coefficients generated by the RC algorithm have significant differences.This fact is verified by the kurtosis and standard deviation of the RC algorithm, which are given in Table 3. In general, correlation coefficients from the NA and RC algorithms are equally distributed, but the NA algorithm produces a higher standard deviation and lower kurtosis, which implies more extreme correlation coefficients than the other algorithms.

**Table 3:** Statistical summary of random correlation coefficients (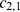 and 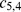).

	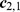			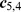		
Statistical measure	NA	RS	RC	NA	RS	RC
Mean	−0.001	−0.0001	−0.0009	0.0004	−0.0004	0.001
Median	−0.0024	−0.0001	−0.0015	−0.0013	−0.0006	0.0011
Standard Deviation	0.5289	0.4079	0.2779	0.5281	0.4086	0.2901
10 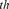 Percentile	−0.7288	−0.5515	−0.3536	−0.7268	−0.5528	−0.3667
90 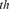 Percentile	0.7301	0.5503	0.3517	0.7297	0.5516	0.3697
Skewness	0.0062	0.0012	−0.0015	0.0027	−0.0018	0.009
Kurtosis	1.9421	2.2551	3.0207	1.9444	2.2496	2.6727

## Discussion

In this paper, we have presented an efficient method to calculate the boundaries of correlation coefficients. We also demonstrated a technique for generating correlation matrices using any bounded random variable distribution within the boundaries of each correlation coefficient. However, this method causes the correlation coefficients to be unevenly distributed. Thus, we incorporated a technique for random reordering to ensure the even distribution of all correlation coefficients. The performance of the proposed algorithm was compared to that of other algorithms. It was shown that the new algorithm could efficiently construct correlation matrices, particularly when the dimension of the matrix was in the range 4–35. In theory, our algorithm should always return valid correlation matrices. However, without setting a threshold factor and using rejection sampling logic, the algorithm exhibited some numerical instability when the dimension became large. It is possible to adjust invalid matrices to form valid ones; this method has been developed in many studies [16], [17], [18]. Therefore, we strongly believe that our new algorithm is useful in the many applications where extreme cases are very important. More importantly, the uniform distribution can be replaced with any bounded distribution.
